# Revolutionizing viral disease vaccination: the promising clinical advancements of non-replicating mRNA vaccines

**DOI:** 10.1186/s12985-023-02023-0

**Published:** 2023-04-07

**Authors:** Xiao Guo, Dongying Liu, Yukai Huang, Youcai Deng, Ying Wang, Jingrui Mao, Yuancheng Zhou, Yongai Xiong, Xinghong Gao

**Affiliations:** 1 School of Basic Medicine, Zunyi Medical University, West No. 6 Xuefu Road, Xinpu District, Zunyi, 563006 Guizhou People’s Republic of China; 2 Department of Hematology, College of Pharmacy, Army Medical University (Third Military Medical University), Chongqing, People’s Republic of China; 3 Modern Medical Teaching and Research Section, Department of Tibetan Medicine, University of Tibetan Medicine, No. 10 Dangre Middle Rd, Chengguan District, Lhasa, 850000 Tibet Autonomous Region People’s Republic of China; 4 Livestock and Poultry Biological Products Key Laboratory of Sichuan Province, Sichuan Animal Science Academy. No, 6 Niusha Road, Jinjiang District, Chengdu, 610299 People’s Republic of China; 5 School of Pharmacy, Zunyi Medical University, West No. 6 Xuefu Road, Xinpu District, Zunyi, 563006 Guizhou People’s Republic of China; 6 Key Laboratory of Infectious Disease and Bio-Safety, Provincial Department of Education, Zunyi Medical University, West No. 6 Xuefu Road, Xinpu District, Zunyi, 563006 Guizhou People’s Republic of China

**Keywords:** Viral disease, Non-replicating mRNA vaccines, Vaccine structure, Clinical research advances

## Abstract

The mRNA vaccine technology was developed rapidly during the global pandemic of COVID-19. The crucial role of the COVID-19 mRNA vaccine in preventing viral infection also have been beneficial to the exploration and application of other viral mRNA vaccines, especially for non-replication structure mRNA vaccines of viral disease with outstanding research results. Therefore, this review pays attention to the existing mRNA vaccines, which are of great value for candidates for clinical applications in viral diseases. We provide an overview of the optimization of the mRNA vaccine development process as well as the good immune efficacy and safety shown in clinical studies. In addition, we also provide a brief description of the important role of mRNA immunomodulators in the treatment of viral diseases. After that, it will provide a good reference or strategy for research on mRNA vaccines used in clinical medicine with more stable structures, higher translation efficiency, better immune efficacy and safety, shorter production time, and lower production costs than conditional vaccines to be used as preventive or therapeutic strategy for the control of viral diseases in the future.

## Introduction

Viral infections have long posed a significant risk to human health security. In particular, since the end of 2019, COVID-19 has spread rapidly across the globe [1]. Given the constant mutation in viruses, the production of conventional vaccines (inactivated vaccines such as CoronaVac [2], live attenuated vaccines such as COVI-VAC (NCT04619628), subunit vaccines such as SARS-CoV-2 recombinant spike protein [3], recombinant viral vector vaccines such as VF17D-vectored SARS-CoV-2 vaccine [4], virus-like particle vaccines such as an adjuvanted plant-based VLP vaccine consisting of S recombinant protein and coronavirus-like-particle technology (https://www.medicago.com/en/media-room/medicago-and-gsk-start-phase-3-trial-of-adjuvanted-covid-19-vaccine-candidate/), DNA vaccines such as AG0301/AG0302 [5], is no longer applicable to the rapid mutation in viruses. mRNA vaccines, on the other hand, are the third generation of vaccines, following inactivated vaccines, live attenuated vaccines, subunit vaccines, and viral vector vaccines. They have the characteristics of rapid response to pathogen mutation, simple production process, and easy scalability [6]. Now they are widely used around the world.

An mRNA vaccine is a chemically modified mRNA molecule that penetrates the cytoplasm and uses its nucleotides for transcriptional expression to produce viral antigens in the cell, thereby triggering a specific immune response in the organism and establishing preventative immunity [7]. mRNA has the theoretical capacity to produce any protein. Due to their economic, safe, rapid, and flexible characteristics, mRNA drugs have a promising future in infectious disease prevention. Nonetheless, single-stranded mRNA is unstable and will be identified and degraded by pattern recognition receptor (PRR) shortly after entering the organism [8–10], which has been a stumbling block for the industry for many years. As a result, the development of mRNA vaccines has been a huge challenge. Several techniques have been used to produce more stable mRNAs as biotechnology develops and matures. First, synthetic structure-modified RNAs are used to replace natural RNAs to synthesize mRNAs that can evade recognition by the body's immune system as "non-self" components, and be eliminated such as pseudouridine (Ψ) [11] used in mRNA-1273. Secondly, 5' cap structures, 3' poly(A) tails, and UTR sequences can also stabilize mRNA and thus increase translation efficiency, such as optimization in these regions used in BNT162b2 [12]. Further, advanced new formulation techniques like lipid nanoparticles can successfully protect mRNA and stimulate immune responses [13], such as LNP used in mRNA-1388. Based on the recent rapid development of COVID-19 mRNA vaccines and their remarkable efficacy in clinical applications [14], driving the development of the entire mRNA vaccine industry, this paper reviews existing mRNA vaccines with potential clinical applications for the treatment of viral diseases. The aim is to provide a theoretical reference for the research of mRNA vaccines for viral diseases with clinical applications.

## mRNA vaccine optimization

mRNA vaccines are now categorized into two major groups: self-amplifying mRNA (SAM) vaccines and non-replicating mRNA vaccines. Based on the exceptional research results in non-replicative mRNA vaccines for viral infections, we are concentrating on the advances of structural research on non-replicative mRNA vaccines.

### 5’ Cap

5’ cap is a unique sequence composed of 7-methylguanosine located at the 5’end of eukaryotic mRNA. 5'cap protects the 5' end of the mRNA sequence from nuclease hydrolysis and enhances the mRNA's stability. It is recognized and bound by the eukaryotic translation initiation complex cap-binding protein (eIF4E) in vivo, which enhances translation efficiency by recruiting the 40S ribosomal subunit [15].

There are three primary 5' cap structure varieties: cap0, cap1, and the anti-reverse cap analog (ARCA). The cap0 structure (m7GpppXpYp structure) is the most common cap structure; however, the analogs of this cap structure frequently bind to the mRNA sequence in reverse throughout the process, leading to the formulation of mRNA heterodimers [16]. To address this problem, ARCA was developed, which is modified at the C2 or C3 position and provides higher translation efficiency of ARCA cap mRNAs compared to conventional caps [12, 17]. In addition, it was demonstrated that the addition of donor methyl S-adenosylmethionine (SAM) to cap0 formed a cap1 structure (m7GpppXmpYp). mRNAs with the cap1 structure could still be translated after being recognized by the PRR.

**Fig. 1:**
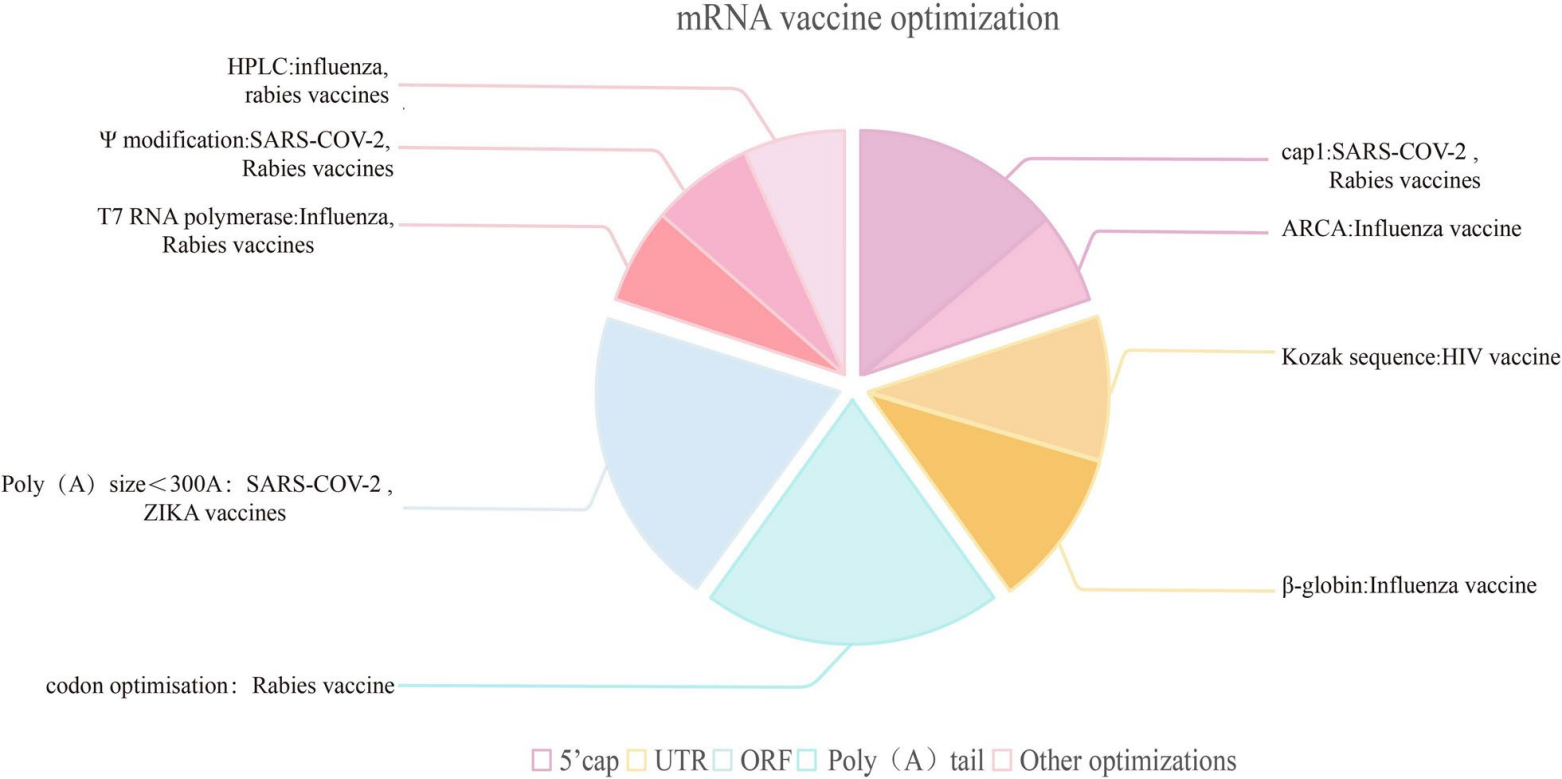
The types of structural optimization in non-replicating mRNA vaccines against viral diseases. The optimization mainly contains 5’Cap (The fan of the Purple border), UTR (The fan of the Yellow border), ORF region (The fan of the Cyan border), Poly(A) tail (The fan of the Grey border), and other optimizations (The fan of the Pink border). The purple-edged sector shows the structural modification of 5’cap, mainly including cap1 (m7GpppXmpYp) and ARCA. Cap1 is widely used to develop mRNA vaccines, such as SARS-COV-2, rabies vaccine, etc. ARCA is relatively less used, and is used in influenza vaccines, etc. The yellow-edged sector shows the structural modifications of the UTR region; the main modifications are Kozak sequence (GCCGCCATG), and β-globin. Kozak sequence is used in HIV vaccines and β-globin is used in influenza vaccines. The cyan-edged sector are structural modifications of the ORF region, mainly for codon optimization, such as GC enrichment, for applications to rabies vaccines. The grey-edged sector is the Poly(A) tail, which mostly keeps the mRNA structure stable when its length is less than 300A, and is used in the SARS-COV-2 vaccine and ZIKA vaccine, and etc. The pink sectors indicate other structural optimizations: HPLC purification, pseudouridine (Ψ) modification, and T7 RNA polymerase. HPLC purification is used in influenza vaccines, rabies vaccines. Pseudouridine (Ψ) modification is widely used in the development of mRNA vaccines, including SARS-COV-2 vaccines, and rabies vaccines and so on. T7 RNA polymerase is also widely used for influenza vaccines, rabies vaccines, etc.

In contrast, mRNAs with cap0 or without cap did not translate correctly, and this structure resulted in nearly 20-fold higher levels of translated protein expression compared to cap0 [18]. The therapeutic mRNA encoding the HIV-1 monoclonal antibody was designed by Pardi et al. to improve the translation efficiency of mRNA by in vitro transcription followed by 5'cap1 capping of RNA using the m7G capping kit with 2'-O-methyltransferase. The result was 30 μg of mRNA transcribed to produce 165 μg/ml of mouse antibody within 24 h [19]. Currently, due to the high stability of cap1, its translation efficiency, and the maturity of the production technology, almost all mRNA vaccines entering clinical studies have adopted this structure, including mRNA-1273, BNT162b2, mRNA-1944, CV7202 and so on (Fig. 1) [20–22].

### Untranslated region (UTR)

An untranslated region (UTR) is located upstream and downstream of the mRNA sequence that does not encode a translated sequence and can be classified into 5’UTR and 3’UTR according to its location [23]. This region is associated with the mRNA stability and translation [24, 25].

The 5’UTR directly affects the efficiency of mRNA translation by binding to the translation initiation complex. A stable 5'UTR secondary structure inhibits the binding of ribosomes to mRNA, whereas a short and incompact 5'UTR structure is more helpful in improving the mRNA translation efficiency [12, 26, 27]. The 5'UTRs of α- and β-globin mRNAs are found in many in vitro-transcribed (IVT) mRNA, and they contain sequence features that enhance the translation and stability of mRNA [12, 23]. BioNTech uses this strategy extensively in the vaccines it develops. In addition, the Kozak sequence is a specific segment of the sequence located at the junction of the 5'UTR and the open reading frame, which can play a key role in targeting the expression of a specific protein independent of the 3'UTR [28]. Using an optimized Kozak sequence (K2 sequence—GCCGCCATG) enhances the stability of the mRNA [20].

The 3'UTR controls translation through binding to RNA binding protein (RBP) [29] and is a sequence that centers instability factors in mRNA. Therefore, unstable regions, such as AU-enriched and GU-enriched regions, should be avoided while synthesizing 3'UTRs. It was found that the 3'UTR may also operate as a polyA-tail-polyA-tail binding protein complex to isolate the buffer when the ribosome approaches the stop codon [30]. In this regard, introducing stabilizing elements during the synthesis of 3'UTRs could also dramatically improve the stability of mRNA and prolong its half-life [12]. Furthermore, 3'UTRs possessing repetitive γ-globin or β-globin can considerably boost the translation efficiency and persistence of the protein (Fig. 1) [31, 32].

### Optimization of open reading frame (ORF)

The ORF sequence is the coding region of the mRNA. mRNA's biological activity is mainly determined by the combination of its primary and secondary structures, which are altered by variations in ORF sequences[33]. So this region is crucial for translation efficiency [34]. mRNAs are significantly more stable and degraded at a reduced rate after codon optimization [35]. Increasing the ratio of G and C bases in the sequence can enhance the stability of mRNA sequences and improve the translation efficiency. Studies have shown that GC-rich genes are expressed several to over 100 times more efficiently than GC-poor genes [36]. Rabies Virus mRNA vaccines designed by Schnee et al. have optimized the GC content of the ORF region and produced vaccines with high stability and translation efficiency [37]. This strategy is also used in the mRNA vaccine against influenza A designed by Benjamin Petsch et al. and in the various vaccines developed by Moderna (Fig. 1) [38].

### Poly(A) tail

Poly(A) sequences can slow down the degradation process of mRNA and improve its stability. Consequently, the half-life of mRNA is extended, and it’s in vivo translation efficiency is increased [35, 39]. Current studies have shown that the translation efficiency of mRNA increases with increasing Poly(A) size in the detectable range (300 A) [40]. For instance, in DC2.4 cells, mRNAs with poly(A) tails consisting of 148 A in length were translated more effectively than those with tails that were 120 A in length (Fig. 1) [41]. In addition, the linear plasmid vector system, pEVL, developed by Grier et al., has allowed the addition of a poly(A) of 500 nucleotides at the 3' end. A follow-up investigation will determine the Poly(A) length that produces the optimum mRNA stability.

### Other optimizations

#### Base modifications

The most prevalent modifications are N6-methyladenosine (m6A), N1-methyladenosine (m1A), inosine (I), pseudouridine (Ψ), 5-methylcytosine (m5C), 5-hydroxymethylcytosine (hm5C), N6,2'-O-dimethyladenosine (m6Am), 7-methylguanosine (m7G), and N4-acetylcytosine (ac4C) [42–44]. The pseudouridine (Ψ) modification is the most prevalent base sequence modification [45]. Substituting pseudouridine(Ψ) for uridine diminishes the stimulation of Toll-like receptors. So it decreases the immunogenicity of mRNA, evades clearance by the body's immune system, increases the dose of effective mRNA, and thereby enhances delivery efficiency. Experiments have demonstrated that sea renal luciferase mRNA containing is translated 10 times more in 293 cells than unmodified mRNA [46]. BioNTech currently uses the Pardi-designed mRNA technology platform, which includes pseudouridine (Ψ) modifications such as BNT162b2, BNT113, and so on [47]. Currently, the majority of mRNA vaccines entering clinical trials contain this change (Fig. 1).

#### mRNA purification

It was shown that contaminant short RNAs and double-stranded RNAs (dsRNA) in mRNA increase the production of type I Interferon (IFN) and pro-inflammatory cytokines [48]. Therefore, mRNA must be purified following synthesis. Commonly used to purify mRNA is High-Performance Liquid Chromatography (HPLC) [49], and purified mRNA can effectively lower its immunogenicity. Most newly developed mRNA vaccines have been purified by HPLC (Fig. 1) [23]. However, the high time and cost of HPLC purification limit the scale-up of mRNA vaccine production [50, 51]. Baiersdorfer presented a cellulose-based chromatographic approach for eliminating dsRNA contaminants from IVT mRNA with different lengths and nucleoside compositions. Furthermore, the purity and biological activity of mRNA purified by this approach are equivalent to HPLC-purified mRNA [51]. This simple, low-cost purifying method has the potential to be used in large-scale manufacturing.

#### New in vitro transcriptase

T7 RNA polymerase (T7RNAP), is one of the simplest enzymes to catalyze RNA synthesis and is the primary gene product of the T7 phage. It is being employed for mRNA in vitro transcription. T7RNAP consists of an N-terminal domain (residues 1–325) and a polymerase domain (residues 326–883) [52]. It has been demonstrated that the T7RNAP method can be utilized to add new components to the mRNA designating site [53], such as adding new stabilizing sequences to enhance the stability of the mRNA (Fig. 1). However, the production of dsRNA by T7 transcriptase during IVT can cause adverse reactions in the organism. Existing vaccination technologies eradicate dsRNA using purifying techniques such as HPLC, but this is a time-consuming and costly process. To solve this issue, it has been demonstrated that firmly coupling promoter DNA and T7 RNA polymerase on magnetic beads induces promoter binding, and transcription initiation improves mRNA purity [54]. A recent study reported that a mutant enzyme (T768) based on the T7 enzyme RNA polymerase capped efficiently and extensively reduced the amount of dsRNA, following HPLC purification [55]. The amount of dsRNA in mRNA transcribed by another in vitro transcriptase (VSW-3) encoded by a bacterial gene product from high salt is approximately less than 1/10 or even less than the dsRNA formed by transcription of the T7 enzyme [56]. In addition to improving mRNA purity, mutants of T7 transcriptase, such as a single mutation (S43Y), considerably improve transcription efficiency [57]. It is believed that more RNA polymerase will be developed in the future, which is another direction of mRNA transcription optimization advancement.

In addition, delivery systems can also be used to improve delivery efficiency and thus increase mRNA expression [58], with lipid nanoparticles (LNP) [59], being the most commonly used delivery system. Subcutaneous, intramuscular, and intradermal injections of mRNA-LNP can continue to translate at the injection site for 10 days [60]. However, novel delivery systems have also demonstrated great delivery advantages. For example, subcutaneous injection of hydrogel-encapsulated ovalbumin mRNA (mOVA) provides sustained release of mOVA for at least 30 days [61]. Thermal stable nanoparticles can store mRNA for at least 6 months at 2–8 °C without reducing immunogenicity [62]. Lyophilized nanoparticles can be stored at room temperature for 12 weeks [63].

A vaccine often has three or more of these optimizations. For example, the Pardi-designed mRNA technology platform currently used by BioNTech and the RNActive platform developed by CureVac (EP1857122 and WO2012019780A1) both use cap1, GC enrichment, pseudouridine (Ψ) modifications, and others [47].

## Recent clinical research advances in mRNA vaccines in viral diseases

### Preventive vaccines

#### mRNA vaccines for RNA viruses

##### SARS-CoV-2 vaccine

**Fig. 2:**
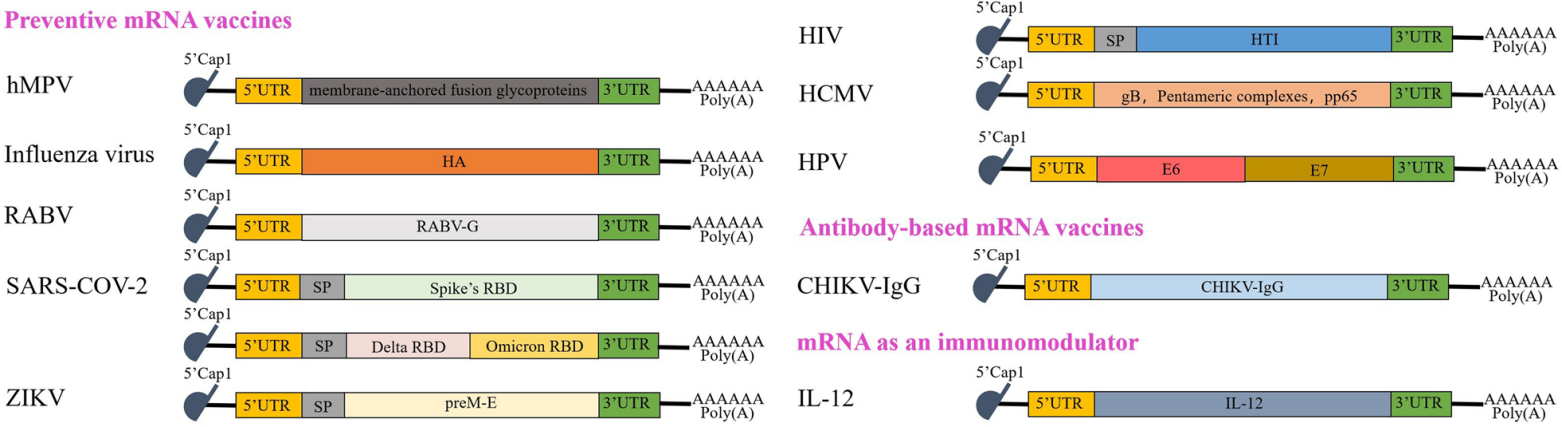
Structures of non-replicating mRNA vaccines entering clinical trials against viral diseases. The structures of non-replicating mRNA vaccines contain 5’Cap, 5’UTR, ORF region, 3’UTR, and Poly(A) tail. The main difference between the vaccine structures is the material encoded in the ORF regions. This article presents three kinds of mRNA vaccines: preventive mRNA vaccines, antibody-based mRNA vaccines, and mRNA as an immunomodulator. In preventive mRNA vaccine, vaccines against RNA viruses include human metapneumovirus (hMPV), influenza virus (IAV), rabies virus (RABV), SARS-COV-2, ZIKA virus (ZIKV), human Immunodeficiency Virus (HIV), human cytomegalovirus (HCMV) and human papilloma virus (HPV). mRNA vaccines encoding antibodies only contain CHIKV-IgG against CHIKV. mRNA vaccines encode cytokines as an immunomodulator, such as IL-12, which activates the immune response

SARS-CoV-2 is a single-stranded positive-stranded RNA virus (+ ssRNA) of the genus *β-coronavirus* in the *Coronaviridae* family [64]. It is the etiologic agent responsible for the ongoing pandemic of Corona Virus Disease 2019 (COVID-19) [65], in which patients present with an insidious, persistent infection that can cause symptoms such as pneumonia and, in severe cases, death [66, 67]. It has infected more than 600 million people worldwide, of which the number of deaths has exceeded six million (https://coronavirus.jhu.edu/map.html). The SARS-CoV-2 spike glycoprotein (Spike, S) attaches to the host receptor and is the primary immunogen of the virus [68], and its receptor binding region(Receptor Binding Domain, RBD) serves as the main coding region for mRNA vaccines (Fig. 2).

Two novel mRNA vaccines against SARS-CoV-2 (mRNA-1273 and BNT162b2) have been approved for urgent clinical use worldwide. BioNTech company designed the BNT162b2 vaccine encoding RBD and delivered using LNP to trigger CD4^+^ helper type 1 T cell (Th1) responses, produce large amounts of Interferon Gamma (IFN-γ) and trigger interleukin 2 mediated CD8^+^ cytotoxic T-cell responses [69]. In a randomized, single-blind clinical trial of BNT162b2, participants were administered two 30 μg doses of vaccine or placebo. The vaccine efficacy was 96.2% from 7 days to 2 months after the second dose, 90.1% from 2 to 4 months after the second dose, and 83.7% from 4 months after the second dose [70]. BNT162b2 is 95% effective in preventing SARS-CoV-2, according to Phase III clinical trials done across different countries [14]. Most adverse effects were injection site pain, fatigue, or headache. Moderna's mRNA-1273 encodes the SARS-CoV-2 spike-in (S) protein [71], and its phase III clinical trial demonstrated that the mRNA-1273 vaccine was 94.1% effective relative to the placebo group and that antibodies would be sustained for 6 months following secondary immunization [72]. It could trigger effective neutralizing activity. In a comprehensive comparison of four vaccines, mRNA-1273, BNT162b2, NVX-CoV2373, and Ad26 COV2.S, Zeli Zhang et al. found that mRNA-1273 could activate more CD4^+^ T cells and produce more memory cells than BNT162b2. At the same time, all immunogenicity indexes of the two mRNA vaccines were higher or equal to those of the other two conventional vaccines [73].

Due to the emergence of different mutants in SARS-CoV-2 (Beta, Gamma, Delta, and Omicron) [74], vaccine companies, represented by Curevac, have started to focus on the development of next-generation mRNA vaccine candidates [75, 76]. The omicron variant has the highest potential for immune escape, making several inactivated vaccines significantly less effective [77]. To address this phenomenon, George F. Gao's team designed dimeric mRNA vaccines encoding both Delta-Omicron strains of RBD (Fig. 2) [78]. They demonstrated that dimeric vaccines increased antigen expression and induced higher neutralizing antibody titers compared to mRNA vaccines encoding only Alpha or Omicron.

##### Zika virus vaccine

Zika virus (ZIKV) is an enveloped, positive-stranded RNA virus (+ ssRNA) of the *Flavivirus* genus and *Flaviviridae* family. The symptoms of ZIKV infection include headache, rash, conjunctivitis, and myalgia [79]. mRNA vaccines encoding nucleoside modifications of the prM-E protein, designed by Richner's team in close collaboration with Moderna, produced high neutralizing antibody titers in animal tests [80]. Among them, AG129 mice that received two doses of 2 μg or 10 μg IgE sig -prM-E LNP vaccine as well as mice that received a single 10 μg dose, survived the infection (Fig. 2). In contrast, serum neutralization titers in C57BL/6 WT mice trials peaked 4 weeks after booster immunization and remained elevated for 18 weeks after the initial vaccination. In addition, the team attacked post-pregnant mice with a lethal dose of a heterologous African ZIKV strain via the subcutaneous route after vaccination. Maternal mice immunized with prM-E mRNA LNP had significantly lower levels of viral RNA in maternal, placental, and fetal tissues relative to placebo controls. Most fetuses showed no evidence of transmission. Thus, the team demonstrated that pre-gestational ZIKV vaccination prevents placental and fetal infections [81]. Following the favorable results of this preclinical trial, Moderna evaluated a phase 1 clinical trial using a nucleoside-modified mRNA vaccine encoding the Zika virus prM-E protein (mRNA-1325) in 90 subjects aged 18–49 years from December 2016 to July 2019, but the results are not yet available.

Antibody-dependent enhancement (ADE) is present in the disease of several flaviviruses such as Zika virus, dengue virus, and West Nile virus [82, 83]. Some researchers proposed designing a vaccine expressing T-cell antigenic epitopes to stimulate T-cell differentiation and development for a protective therapeutic effect. Roth et al. created an mRNA vaccine targeting the T-cell epitope of the NS protein [84]. In vivo tests on mice demonstrated its potent immunogenicity and protective efficacy. This may be a new path for future Flaviviridae mRNA vaccine development.

##### Ebola virus vaccine

Ebola virus (EBOV), a genus of *Ebolavirus* in the *filoviridae* family, is a single-stranded negative-stranded RNA virus. Like most other RNA viruses, it is prone to mutations during replication [85]. Humans and other primates are susceptible to Ebola hemorrhagic fever (EBHF), a severe form of hemorrhagic fever, caused by a highly pathogenic virus [86]. Common early symptoms in patients include fever, general malaise, loss of appetite, vomiting, diarrhea, headache, and abdominal pain [87], and the case fatality rate can be as high as 90% [88]. Meyer et al. designed 2 mRNA vaccines encoding EBOV glycoprotein (GP). Vaccine A encodes full-length EBOV GP, and its structure contains 5' UTR + EBOV GP + 3' UTR + Poly A, while in vaccine B, the signal peptide of GP is exchanged with that from human immunoglobulin κ (Igκ). Both vaccines are administered to guinea pigs after encapsulation using LNP [89]. The experimental results showed that both vaccines successfully protected guinea pigs from lethal EBOV infection, and neither showed clinical signs of disease. After one dosage, vaccine B produced stronger GP-specific IgG and EBOV-neutralizing antibodies than vaccine A. The neutralizing antibodies produced by vaccine B were maintained beyond day 21, whereas the neutralizing antibodies produced by vaccine A were not maintained for a more extended period. After the second dose, EBOV-neutralizing antibodies were also detected in vaccine A, while antibody levels in vaccine B were further increased. The results indicate that a single dose of vaccine B induced a robust immune response against the virus in guinea pigs. Therefore, the addition of the IgK signal peptide to the mRNA structure can potentially improve the mRNA's translation efficiency. Although no registered mRNA vaccine against Ebola has yet been used in clinical trials, it is believed to enter clinical trials soon based on the good results of pre-clinical trials.

##### Influenza virus vaccine

Influenza viruses are single-stranded negative-stranded RNA viruses of the genus *Influenzavirus* of the *Orthomyxoviridae* family and have caused several pandemics in history. Influenza viruses are typed based on two glycoproteins, hemagglutinin (HA) and neuraminidase (NA), for which mRNA vaccines are designed (Fig. 2) [38, 90].

Before 2012, Petsch et al. designed an mRNA vaccine against full-length HA of influenza A (H1N1, H3N2, and H5N1) [38], and two intradermal injections of 80 µg H1 mRNA vaccine-induced antigen-specific T cell responses in mice, and in ferrets. The immunogenicity of the 80–250 µg H1 mRNA vaccine was comparable to that of licensed inactivated viral vaccines. 250 µg mRNA induced a strong antibody response in domestic pigs, meeting the serological standards for licensed seasonal influenza vaccines. However, the research of the above early mRNA vaccine platform in the modification and delivery system is not yet mature. Therefore, Bahl et al. developed base modified (such as pseudouridine and 5-methylcytidine substituted uridine nucleosides) HA mRNA nanoparticle vaccines against H10N8 and H7N9 viruses [91], and performed antibody titer assays and attack protection experiments in mice, ferrets, and non-human primates, respectively. Vaccinations protected individuals with intact mRNA structures that achieved hemagglutination inhibition (HAI) titers above the detection threshold. They also demonstrated that both intramuscular and intradermal injections were equivalent and generally required secondary immunization at species-specific doses. Feldman et al. conducted a randomized, double-blind phase 1 clinical trial targeting H10N8 and H7N9 [92]. The trial results showed that for H10N8, an intramuscular dose of 100 μg induced HAI titers ≥ 1:40 up to 100% and microneutralization (MN) titers ≥ 1:20 up to 87.0%. For H7N9, intramuscular doses of 10, 25, and 50 μg achieved HAI titers ≥ 1:40 in 36.0%, 96.3%, and 89.7% of participants, respectively. The percentage of MN ≥ 1:20 was 100% in both the 10 and 25 μg groups and reached 96.6% in the 50 μg group. The results indicated that the intramuscular administration of H10N8 and H7N9 mRNA vaccines exhibited good safety and reactogenicity, and no serious vaccine-related adverse events were reported. Furthermore, the observed effects were consistent with expectations.

##### Rabies virus vaccine

Rabies virus (RABV), a genus of *Lyssavirus* in the family *Bomboviridae*, is a single-stranded negative-stranded RNA virus that causes an infectious disease in humans and animals. Rabies primarily affects the neurological system of humans and animals, has a long incubation period, and once it develops, the case fatality rate reaches 100% [93]. Despite the availability of effective vaccination, rabies kills roughly 50,000 people worldwide each year [94]. Most of these infections occur in developing countries, which may be related to developing countries economic and technological levels.

Schnee et al. induced effective neutralizing antibodies in mice and domestic pigs by an optimized complex of mRNA encoding the non-replicating form of rabies virus glycoprotein (RABV-G) with protamine by intradermal injection (Fig. 2) [37]. RABV-G mRNA induced specific CD4^+^ and CD8^+^ T cells and higher amounts of CD4^+^ T cells than existing licensed vaccines HDC or Rabipur. The RABV-G mRNA vaccine was as effective as HDC in establishing protective immunity against rabies virus in mice and preventing infection. The RABV-G vaccine elicited high neutralizing antibody titers and high immunogenicity in newborn piglets. Subsequently, the phase I clinical trial results showed that this mRNA vaccine (CV7201), administered intradermally using a needle-free injection device, was most effective [95]. 40 of the 45 (89%) participants receiving 80 μg or 160 μg produced antibodies after vaccination, of which 32 (71%) produced antibody titers of ≥ 0.5 IU/mL (WHO set threshold). This shows that the strength and duration of the immune response of this vaccine still need to be improved. Although the local adverse reactions caused by this vaccine were mild or moderate, the incidence was high. Further modification and purification of the mRNA are still needed to reduce the stimulation of the mRNA on the body's immune response in later trials. In addition, the mRNA rabies virus vaccine designed by Stitz et al. demonstrated in mouse experiments that the mRNA vaccine was lyophilization with trehalose and re-solubilized in the buffer before injection. Moreover, this lyophilized vaccine could be stored at 70 °C for several months without affecting the protective power of the vaccine [96]. The issue of mRNA's low heat resistance has been efficiently resolved, which could assist in minimizing the rabies infection rate in developing nations.

#### mRNA vaccines for Retrovirus

##### Human immunodeficiency virus vaccine

Human Immunodeficiency Virus (HIV) is a genus of *lentivirus* in the *Retroviridae* family. It attacks the human immune system, especially attacking CD4^+^ T cells, making it vulnerable to tumors or other infectious diseases, with a very high lethality rate. HIV is first formed into an RNA/DNA heterogeneous double helix by the action of reverse transcriptase. RNA strands are then broken down by ribonuclease and the polymerase of reverse transcriptase is active on the DNA strand to complete a complementary DNA strand, forming a double helix DNA molecule. Eventual integration into the host cell genome by integrase [97, 98]. The integrated gene is passed on to offspring cells as the parental generation divides, making it difficult to remove and treat. There is no approved HIV vaccine against Acquired Immune Deficiency Syndrome (AIDS). Several dendritic cells (DC)-delivered HIV mRNA vaccines are currently being evaluated in clinical trials. However, despite their efficacy in inducing humoral and cellular immune responses, they have not achieved considerable clinical success, and a viable, functional vaccine has not yet been developed [99]. Gag genes encode structural proteins of the core (P24, P7, P6) and matrix (P17). Rev genes encode Rev proteins that ensure the correct messenger and genomic RNA processing for export from the nucleus to the cytoplasm. Vpr proteins are involved in cell cycle arrest, and Nef proteins have multiple functions, including cell signaling and downregulation of CD4 receptors [97]. Jacobson et al. designed an mRNA vaccine encoding Gag, Rev, Vpr and Nef proteins delivered by DC in a randomized, double-blind clinical trial in 54 test subjects. Although no significant antiviral effect was observed, a more intense HIV-specific CD8^+^ T cell response was observed in the experiment [100], which opens the possibility of clinical translation of mRNA vaccines as essential HIV therapeutics in the future. In addition to the commonly used DC delivery system, Zhao et al. designed an HIV-encoded Gag protein mRNA vaccine delivered via polyethyleneimine stearate (PSA), which was shown to significantly improve mRNA transfection efficiency and cellular uptake efficiency while maintaining relatively low cytotoxicity in a mouse assay. In addition, large amounts of CD80 were induced by this delivery mechanism [101]. They offer a novel approach to the creation of future HIV mRNA vaccines. Pardi et al. designed an HIV-1 mRNA vaccine containing m1Ψ-modified nucleosides encoding the light and heavy chains of VRC01 using LNP. The results of preclinical trials showed that viral load was below the minimum detectable level in all mice treated with 30 μg or 15 μg of VRC01 mRNA-LNP before the HIV-1 attack (Fig. 2). No less than 600 μg VRC01 protein subunit vaccine exerted a protective effect [19], demonstrating its protective and efficient nature. The IVAI-sponsored IAVI G001 recombinant subunit vaccine uses eOD-GT8 60mer as the immunogen. It produced potent IgG in 35 of 36 vaccinated subjects. Based on the promising trial results, Moderna partnered with IVAI to develop an mRNA vaccine encoding eOD-GT8 60mer (mRNA-1644) and began Phase I clinical trials in May 2022, which is still in test [102, 103].

#### mRNA vaccines for DNA viruses

##### Epstein-Barr virus vaccine

Epstein-Barr virus (EBV), a genus of *lymphophilic* viruses of the *gamma herpesviruses* subfamily, infects more than 90% of people worldwide during their lifetime [104], but the infection is frequently unrecognized. EBV, one of the most important tumor-causing viruses, can cause a variety of diseases with regional differences [105]: infectious mononucleosis occurs mainly in Europe and North America and usually affects adolescents or young adults; in equatorial Africa, Burkitt's lymphoma is associated with EBV infection; in Taiwan, southern China and Southeast Asia, this virus frequently causes nasopharyngeal and laryngeal cancers. In addition, EBV can cause gastric cancer, Hodgkin's lymphoma, and diffuse large B-cell lymphoma. However, there is no effective vaccine against this virus. mRNA-1189, a vaccine designed by Moderna against EBV, encodes the mRNA of four proteins, EBV gp350, gH/gL, gB, and gp42, and is undergoing phase I clinical trials. An mRNA vaccine encoding EBV-LMP2 has been successfully developed at West China Hospital of Sichuan University (Application No. 202010687809.1), which incorporates the intracellular sequence of the MHC I molecule into the vaccine structure. This will provide reference data for subsequent clinical trials.

##### Human cytomegalovirus vaccine

Human cytomegalovirus (HCMV), a genus of *cytomegalovirus* belonging to the *herpesvirus* subfamily, can cause glioblastoma (GBM) [106], infectious mononucleosis [107], and congenital deafness in children [108]. GBM is one of the most aggressive and therapeutically challenging tumors in adults and children. Diken et al. demonstrated that DCs selectively take up ribonucleic acid and induce an anti-tumor T-cell response, a finding that could be used to treat tumors [109]. Batich et al. designed a vaccine against the cytomegalovirus (CMV) protein pp65 in GBM. They conducted three independent clinical trials using CMV-specific DC-loaded mRNA vaccine in newly diagnosed GBM patients [110]. In the first, a blinded, randomized phase II clinical trial (NCT00639639), over one-third of patients in this cohort were free of tumor recurrence within five years of diagnosis, according to follow-up data. In the second, the survival rate of 36% five years after diagnosis was counted for the above trial. The third clinical trial (NCT02366728) showed increased migration of the DC vaccine to draining lymph nodes. The consistency of the three CMV clinical trials increases confidence in the results and provides good evidence of the potential for vaccine development. Pentameric complex (PC) neutralizes and targets CMV gH/gL/UL128/UL130/UL131A on endothelial, epithelial, and bone marrow cells. Recent studies have shown that pp65 in combination with PC and gB induces a more intense T-cell response in mouse models and that this response is enhanced by immunization with a monovalent mRNA vaccine with pp65 followed by a trivalent mRNA vaccine targeting PC + gB + pp65 delivered by LNP compared to pp65 alone or a trivalent vaccine alone (PC + gB + pp65) better (Fig. 2) [111]. It has completed phase I and II clinical trials, and phase III clinical studies are underway. It is the most promising HCMV preventive vaccine currently available for clinical use.

##### Human papilloma virus vaccine

Human papilloma virus (HPV), genus *Papilloma Vacuolavirus A*, and family *Papillomaviridae* can cause skin warts, head and neck squamous cell carcinoma (HNSCC), and cervical cancer in humans. Cervical cancer and squamous cell carcinoma of the head and neck are the most serious, and HPV type 16 and HPV type 18 are the most pathogenic subtypes of cervical cancer [112]. The immunogenicity of HPV16 E7 RNA-LPX was investigated by immunizing C57BL/6 mice twice with vaccine or saline, and the results showed that E7 RNA-LPX induced the production of a large number of cytokines and up to 35% of CD8^+^ T cells after the second immunization. In a mouse tumour model, E7 RNA-LPX showed significant anti-tumour effects against tumours induced by TC-1 cells and had some memory effects[113]. BioNTech's LNP-encapsulated mRNA vaccine BNT113 (HPV16 E6/E7 RNA-LPX) encoding HPV16 E6 and E7 proteins has entered clinical trials have been conducted and published data so far indicate that the vaccine has a good safety profile with adverse effects mainly being AEs (pyrexia, shaking/rigors), but no deaths have been reported (Fig. 2).

##### Herpes simplex virus vaccine

Herpes simplex virus (HSV), a genus of *simplexvirus* in the *alphaherpesviruses* subfamily, has two subtypes: HSV-1 and HSV-2. Infection with HSV-2 is connected with more severe illnesses, including genital herpes and cervical carcinoma [114]. Several mRNA vaccine developments against HSV have been carried out, but they are limited to preclinical studies and have not yet entered clinical trials. Awasthi et al. designed gD2, gC2, and gE2 trivalent mRNA-LNP vaccines encoding HSV-2 [115], and the experimental results showed that vaginal cultures were performed on day 4 of HSV-2 infection in mice experiments. The results showed an infection rate of 23% in mice vaccinated with the trivalent subunit vaccine and only 1.5% in mRNA-vaccinated mice. In guinea pigs, the trivalent subunit vaccine completely protected guinea pigs from genital damage, but 50% of the guinea pigs showed genital shedding of HSV-2 DNA. In contrast, the mRNA vaccine not only completely protected guinea pigs from genital damage but also prevented 80% of subclinical genital shedding of HSV-2 DNA. In a subsequent vaccine persistence assay, serum neutralizing antibody titers in the subunit vaccine group decreased 6.2-fold after 8 months compared with the first month, whereas in the trivalent mRNA vaccine group, the decrease was only 2.2-fold [116]. The combined results show that the trivalent mRNA vaccine to protect this viral infection will excel in clinical trials.

### mRNA encoding viral antibody

Monoclonal antibodies have a more significant effect on emergency prophylaxis after viral infection, interruption of transmission, and treatment than vaccine effects [117, 118]. However, conventional approaches for synthesizing monoclonal antibodies have a long cycle and high prices [119] In contrast, mRNA-encoding viral antibodies is an established production method with short manufacturing lead times, cheap costs, and easier production processes. Several studies have shown that a small dose of mRNA vaccine can produce many neutralizing antibodies with better protection and antibody half-life than direct antibody injection [120, 121]. The principle is that the delivery system delivers the mRNA to a specific target cell. Upon entry into the target cell, the mRNA is translated within the cell to produce the corresponding antibody it encodes, which treats the disease [122]. Pardi et al. designed HIV-1 mRNA vaccines encoding VRC01 light and heavy chains. Results from preclinical trials showed viral loads below the lower limit of detection in all mice treated with 30 μg or 15 μg VRC01 mRNA-LNP prior to HIV-1 attack. The 30 μg mRNA produced much higher antibodies in the organism than those produced by the 600 μg VRC01 protein subunit vaccine. This demonstrates the protective and highly effective nature of mRNA encoding antibody vaccines [19].

#### Chikungunya virus vaccine

Chikungunya virus (CHIKV) is a single-stranded positive-stranded RNA virus transmitted by the Aedes aegypti mosquito, with clinical symptoms often manifesting as fever and severe joint pain, but there is no approved prophylaxis or treatment against this virus [123]. Kose isolated the human monoclonal antibody CHKV-24 with strong neutralizing ability from B cells of survivors of natural CHIKV infection and constructed an mRNA vaccine encoding its monoclonal antibody (Fig. 2)[121]. In mouse experiments, an increase in CHKV-24 IgG concentration was observed with increasing doses of mRNA, and subsequent results of attack protection showed that CHKV-24 mRNA treatment completely protected AG129 mice in a lethal attack model when antibody concentrations of 0.5 mg/kg LNP-mRNA were reached in mice. Moderna's two mRNA vaccines against chikungunya virus (mRNA-1944 and mRNA-1388) have completed Phase I clinical trials, although neither has published complete data yet. Among them, mRNA-1944, encoding antibodies to chikungunya virus (CHKV-IgG), induced 2.0, 7.9, and 10.2 µg/mL CHKV-IgG in 38 subjects 24 h after a single intravenous dose of 0.1, 0.3 and 0.6 mg/kg, respectively, with peak CHKV-IgG production within 48 h and a half-life of about 69 days [21, 124]. Most of these reported adverse reactions were Grade 1 events, none serious, and the safety profile was good.

In addition to the Chikungunya virus vaccine, preclinical trials of mRNA vaccines encoding the corresponding antibodies have been completed in other viruses such as the Influenza A virus vaccine, SARS-CoV-2 vaccine ZIKV, Respiratory Syncytial Virus (RSV), and Human metapneumovirus (hMPV) which have obtained promising results [125–128].

### mRNA as an immunomodulator

mRNA immunomodulators are mRNAs encoding specific proteins such as, cytokines, that are injected into tumors and act to activate the immune response. mRNA mixtures designed by Hotz et al. encoding Interleukin-12 (IL-12) single chain, type I Interferon alpha (IFN-α), granulocyte–macrophage colony-stimulating factor (GM-CSF), and IL-15 sushi (sushi structural domain of the IL-15 receptor α chain fused to IL-15), altered the intra-tumor microenvironment, enhanced CD4^+^ and CD8^+^ T cell immunity in mice, and improved survival in tumor-tested mice [129]. IL-12, produced and secreted extracellularly by antigen-presenting cells, can alter the function and fate of many cell populations that determine disease prognosis and activate the immune response of tumor cells. mRNA vaccine encoding IL-12 (NCT03946800), prepared by MedImmune in association with Moderna, has entered a phase 1 clinical trial (Fig. 2) [130] and showed an immune response in 2 out of 10 patients with good safety and tolerability. Currently, mRNA immunomodulators are mainly used to treat tumors. However, immunomodulators have played an important role in traditional vaccine studies. Therefore, we believe that mRNA immunomodulators could be used in the future for the treatment or prevention of viral diseases in immunocompromised patients, as well as for oncogenic virus prophylaxis and treatment.

## Discussion

Emerging and sudden-onset infectious diseases is one of the major challenges facing public health today. Moreover, the development of mRNA vaccines has made it possible to effectively control various emerging and sudden-onset infectious diseases, resulting in the rapid development and widespread application of mRNA vaccine technology during the SRAS-CoV-2 pandemic. This review provides an overview of the technical optimization and clinical application of the current production stage of non-replicating antiviral mRNA vaccines to provide a reference for the subsequent development of new antiviral mRNA vaccine development. Thereby promoting the development of the level of prevention and treatment of clinical viral diseases.

Technical optimization at the production stage includes structural optimization (cap addition, A addition, UTR sequence selection, base modification, codon optimization, incorporation of signal peptides [89]) (Fig. 1), purification of mRNA products, and development of new RNA polymerase and delivery systems to increase the stability, translation efficiency, and delivery efficiency of mRNA [62, 63], to enhance immune efficacy and safety (reducing the occurrence of adverse reactions). There is also optimization of the mRNA mechanism of action. When an mRNA vaccine enters the body, it stimulates the release of exosomes, which contain microRNA (miRNA). This inhibits type I IFN signaling, exacerbates disease, and activates chronic viral infection [131]. Therefore, reducing the secretion of miRNA or accelerating its degradation would be beneficial in reducing the vaccine's adverse effects. In addition, several novel structures of mRNA vaccines have begun to emerge. Self-amplified RNA (saRNA) vaccines maintain the advantages of rapid development, simple manufacturing process, and easy scalability of mRNA vaccines while requiring only low doses to achieve prophylactic effects due to their self-replicating properties [132, 133]. Several vaccines have entered clinical trials and may become important tools for treating viral diseases in the future. Trans-amplified mRNA (taRNA) is an upgraded version of saRNA technology in which the replicase can simultaneously amplify a large number of mRNAs encoding different proteins, allowing more flexibility in developing therapeutic mRNAs [134]. However, taRNA systems have not been systematically studied and need further exploration. Circular RNA (circRNA) vaccines are circular RNAs encoding antigens that produce higher and more persistent antigens and trigger a higher proportion of neutralizing antibodies and a strong Th1-biased immune response compared to conventional non-replicating mRNA vaccines [135]. Therefore, developing novel structural RNA vaccines is one of the future directions.

Preventive and therapeutic mRNA vaccines have been created for clinical purposes against the virus's antigens, antibodies, and host immunomodulatory proteins. Several prophylactic mRNA vaccines against viruses have been approved for commercialization or enrolled in clinical trials (Table 1), with the majority achieving favorable clinical results. For example, due to the changing infectivity and antigenicity of the SARS-COV-2 virus [136], α, β, Delta, and Omicron strains have emerged, and the immune effect of traditional inactivated vaccines, mRNA-1273, and BNT162b2 vaccines has been diminished. Therefore, scientists have rapidly designed multivalent mRNA vaccines that can simultaneously prevent infection with α, β, Delta, and Omicron strains of the SARS-COV-2 virus, which fully demonstrates the advantages of mRNA vaccines in terms of rapid response and immunological efficacy against pathogenic mutations [137].

**Table 1:** Clinical advances in non-replication structure mRNA vaccines of viral diseases

Virus type	Virus name	Product name	Research and development unit	Coded substances	Delivery systems	Delivery site	ClinicalTrials.gov identifier	Phase	Research progress	Publications
RNA viruses	CHIKV	mRNA-1388(VAL-181388)	Moderna	CHKV-IgG	LNP	Intravenous injection	NCT03325075	I	Completed	-
	CHIKV	mRNA-1944	Moderna	CHKV-IgG	LNP	Intravenous injection	NCT03829384	I	Completed	[21]
	hMPV、PIV3	mRNA-1653	Moderna	Membrane-anchored fusion glycoproteins of hMPV and PIV3	-	-	NCT03392389	I	Completed	-
	hMPV、PIV4	mRNA- 1653	Moderna	Membrane-anchored fusion glycoproteins of hMPV and PIV4	-	-	NCT04144348	I	In test	[143]
	H7N9 Influenza virus	VAL-339851(mRNA-1851)	Moderna	H7N9 HA	LNP	Intramuscular injection	NCT03345043	I	Completed	[91]
	H10N8 Influenza virus	VAL-506440(mRNA-1440)	Moderna	H10N8 HA	LNP	Intramuscular injection/Intradermal injection	NCT03076385	I	Completed	[92]
	Seasonal Influenza Virus	mRNA- 1010	Moderna	H1N1, H3N2 HA, Influenza B virus HA	–	Intramuscular injection	NCT04956575	I/II	Completed	-
	Rabies	CV7201	Curevac	Rabies virus G protein	LNP	Intradermal injection	NCT02241135	I	Completed	[95]
	Rabies	CV7202	Curevac	Rabies virus G protein	LNP	Intramuscular injection	NCT03713086	I	Completed	[22]
	Rabies	GSK3903133A	GSK	Rabies virus G protein	LNP	_	NCT04062669	I	Test completed, analysis not yet completed	-
	RSV	mRNA-1345	Moderna	–	LNP	_	NCT04528719	I	In test	-
	SARS-COVID-2	CVnCoV	Curevac	Spike (S) glycoprotein	LNP	Intramuscular injection	NCT04652102	III	Completed	[75]
	SARS-COVID-2	BNT162a1/BNT162b1/BNT162b2/BNT162c2	BioNTech/Pfizer	Spike (S) glycoprotein	LNP	Intramuscular injection	NCT04380701	I/II	In test	-
	SARS-COVID-2	BNT162b2	BioNTech/Pfizer	Spike (S) glycoprotein	LNP	Intramuscular injection	NCT04588480	I/II	Completed	[144]
	SARS-COVID-2	mRNA-1273	Moderna	Spike (S) glycoprotein	LNP	-	NCT04470427	III	Completed	[145]
	Zika	mRNA-1893	Moderna	preM-E with JEV leading sequence	Lipid nanoparticles V1GL	_	NCT04064905	I	Completed	-
	Zika	mRNA-1325	Moderna	prM-E	LNP	_	NCT03014089	I	Completed	-
Retrovirus	HIV	iHIVARNA-01	Massachusetts General Hospital、National Institute of Allergy and Infectious Diseases (NIAID)	HTI	DCs	Inguinal Intra-inguinal injection	NCT02413645	I	Completed	[146]
	HIV	iHIVARNA-01	Massachusetts General Hospital、National Institute of Allergy and Infectious Diseases (NIAID)	HTI	DCs	Intradermal injection	NCT02888756	II	Completed	[147]
	HIV	–	Massachusetts General Hospital	Gag And Nef	DCs	Intradermal injection	NCT00833781	I/II	Completed	[146]
	HIV	mRNA-1644	Moderna/IVA	eOD-GT8 60mer	LNP	Intramuscular injection	NCT05001373	I	In test	-
DNA viruses	HCMV	mRNA-1647/mRNA-1443	Moderna	mRNA-1647: gB, PC pentameric complex; mRNA-1443: pp65	LNP	_	NCT03382405	I	Completed	-
	HCMV	mRNA-1647	Moderna	gB, PC pentameric complex	LNP	_	NCT04232280	II	Completed	-
	HPV	BNT113	BioNTech	E6 and E7	LNP	Intravenous injection	NCT05448885	II	In test	[113]

In terms of mRNA therapy, a variety of mRNA vaccines encoding antibodies to viruses are available in preclinical trials to treat animals infected with the related viruses (such as HIV, SARS-COV-2, chikungunya fever virus, influenza virus, etc.), which have achieved the expected results and have entered clinical trials. In the course of vaccine development, we found that viruses transmitted by arthropods (such as chikungunya virus) and Flaviviridae viruses transmitted by insect vectors (such as West Nile virus, dengue virus, ZIKV virus) usually suffer from antibody-dependent enhancement (ADE) [82, 83]. The monoclonal antibody designed by Kose et al. against CHIKV E2 CHIKV 24, a therapeutic mRNA-LNP vaccine, was effectively expressed in both mouse and non-human primate sera and was effectively prevented CHIKV [121]. Therefore, we speculate that all viruses causing ADE may be treated and urgently prevented by developing antibodies against a single antigenic epitope (monoclonal antibodies). At the same time, antibody-based mRNA vaccines have the advantages of a simple production process, easy scalability, and good immunization effect, which are crucial for future vaccine development.

In general, mRNA vaccines have the advantages of rapid response to pathogenic mutations, a simple production process, easy scalability, and good immunization effect, making them an important tool preventing and treating future viral illness development. However, some adverse effects of mRNA vaccines, such as hepatitis [138], allergic reactions caused by polyethylene glycol (PEG) [139], and viral reactivation [140], still require researchers to improve vaccine production technologies or delivery systems continuously. To date, self-amplified and trans-amplified mRNA vaccines, delivery systems targeting specific tissues (such as targeting the lung [141]), core–shell structured lipopolyplex (LPP) [142] delivery systems, and new in vitro transcriptases (reduced dsRNA production, vsw-3-RP, and T7 RNA polymerase mutants [55, 56]) have emerged, signifying the maturation of mRNA vaccine production technologies.

It is believed that mRNA vaccine production technology will become more mature and effective, with lower adverse effects and faster production. Multivalent mRNA and other forms of RNA vaccines will also gradually enter the public domain and become an important technology for public health services.

## Data Availability

Datasets used and/or analyzed in this study available from the corresponding author on reasonable request.
